# Examining Social Media Experiences and Attitudes Toward Technology-Based Interventions for Reducing Social Isolation Among LGBTQ Youth Living in Rural United States: An Online Qualitative Study

**DOI:** 10.3389/fdgth.2022.900695

**Published:** 2022-06-27

**Authors:** César G. Escobar-Viera, Sophia Choukas-Bradley, Jaime Sidani, Anne J. Maheux, Savannah R. Roberts, Bruce L. Rollman

**Affiliations:** ^1^Department of Psychiatry, School of Medicine, University of Pittsburgh, Pittsburgh, PA, United States; ^2^Center for Enhancing Treatment and Utilization for Depression and Emergent Suicidality (ETUDES), School of Medicine, University of Pittsburgh, Pittsburgh, PA, United States; ^3^Center for Behavioral Health, Media, and Technology, Division of General Internal Medicine, School of Medicine, University of Pittsburgh, Pittsburgh, PA, United States; ^4^Department of Psychology, University of Pittsburgh, Pittsburgh, PA, United States; ^5^Department of Behavioral and Community Health Sciences, Graduate School of Public Health, University of Pittsburgh, Pittsburgh, PA, United States; ^6^Department of Psychological and Brain Sciences, College of Arts and Sciences, University of Delaware, Newark, DE, United States

**Keywords:** social isolation, LGBTQ, social support, social media, qualitative study

## Abstract

**Purpose:**

Lesbian, gay, bisexual, transgender, and queer (LGBTQ) youth living in rural areas who feel isolated are at high risk of depression and suicidality. Given the lack of support in their offline communities, many rural-living LGBTQ youth turn to social media for social support. In this qualitative study, we examined rural LGBTQ youth's social media experiences and attitudes toward technology-based interventions for reducing perceived isolation.

**Method:**

In Spring 2020, we conducted online interviews with LGBTQ youth aged 14-19, living in rural areas of the United States, who screened positive for perceived social isolation (n = 20; 11 cisgender sexual minority, 9 transgender). Interviews examined (1) supportive social media experiences, (2) personal strategies to improve social media experiences, and (3) perspective on potential digital intervention delivery modalities. Data were analyzed using thematic analysis.

**Findings:**

Related to supportive content and interactions, themes included (1) positive representation of and connecting with LGBTQ groups on social media are important; (2) content from people with shared experience feels supportive, and (3) lack of feedback to one's experiences is isolating. Regarding personal strategies to improve social media experiences, themes were (1) selecting platforms to connect with different audiences helps make for a more enjoyable social media experience, and (2) several social media platform features can help make for a safer social media experience. Youth discussed advantages and disadvantages of intervention delivery via a mobile app, social media pages or groups, conversational agents (chatbots), and a dedicated website.

**Conclusion:**

Viewing positive representation of and connecting with LGBTQ groups, content from people shared experiences, and utilizing a wide array of platform features to increase the likelihood of positive connections are key to a positive social media experience among this group. Combining delivery modalities is key to engaging rural-living LGBTQ youth in digitally delivered support interventions to reduce perceived isolation. Our results inform future intervention research and conversations about social determinants of health between providers and rural LGBTQ patients.

## Introduction

Social isolation is a growing public health concern (1, 2) that has been linked to increases in overall mortality, cardiovascular disease, and poorer mental health outcomes, (3) especially depression (4, 5) and suicidality (6–8). Social isolation is “the inadequate quality and quantity of social relations with other people at the different levels where human interaction takes place, which includes individual, group, community, and the larger social environment.” (9) Among youth, risk factors for social isolation include life events and circumstances, such as living in rural areas (10) or identities that do not conform with social and cultural norms, such as identifying as lesbian, gay, bisexual, transgender, or queer (LGBTQ) (11). LGBTQ youth living in rural areas are at higher risk of social isolation and negative mental health outcomes than non-LGBTQ peers (12). and these mental health disparities are even larger compared to urban-living peers (13, 14). Community resilience, community support, and community connectedness protect against social isolation and negative mental health outcomes and could motivate LGBTQ youth to engage in online communities (15, 16, 56–58). However, those resources might not be as available to LGBTQ youth in rural areas, where close-knit communities value familiarity and sameness (17, 18). In addition, the geographic isolation or distance from more diverse urban areas creates significant logistic barriers for developing LGBTQ adolescents to find LGBTQ-specific well-being and support programs in schools and communities, including the lack of safe spaces, community support and visibility, and assistance with their LGBTQ identity development and coming out, as well as culturally competent physical and mental health care (59).

Social media use is ubiquitous among youth (19), in particular LGBTQ youth (20). Rural-living LGBTQ youth may turn to social media to meet others like them, feel connected to a community, or seek information and social support perceived as unavailable in their offline communities (21, 22). Although some research suggests that offline connections may be higher quality than online ones, these may provide valuable companionship for youth who feel socially isolated, increasing their self-esteem and perceived support (23, 24). These characteristics highlight the potential of leveraging social media to deliver interventions that provide information and support to rural-living LGBTQ youth (25). However, social media can also be a conduit for rejection, discrimination, bullying, and other negative experiences, potentially increasing social isolation and risk for negative mental health outcomes (26, 27). While there is research focused on understanding individual- and community-level needs of social support among rural-living LGBTQ youth, (2, 11, 28) little is known about the potential of leveraging social media for interventions seeking to provide support and reduce perceived isolation among this group (29).

The features of social media have deeply transformed the experience of traditional peer interactions (19, 30). For example, the asynchronicity and permanence of social media content and interactions provide a potential for more frequent and immediate social support, but the absence of cues in many of these interactions might reduce the richness of said support. Gaining awareness of and managing these features may pose a steep learning curve for rural-living LGBTQ youth, who are discovering and exploring their sexual and/or gender identity in an environment that might not have the support resources they need, along with other age-related developmental tasks. Indeed, increasing both community support and access to LGBTQ-specific mental health resources are top needs among LGBTQ youth living in rural areas (11, 31). We need intervention tools to assist these youth in enhancing their social media experience (e.g., reducing negative interactions, increasing supportive experiences) and reducing perceived social isolation. These interventions are not currently available (25).

Digital intervention development that tailors the delivery modality to incorporate the needs, experiences, and preferences of potential users and combines digital and human support increases the likelihood of user uptake and engagement with said interventions (32, 33). While previous research examined how LGBTQ youth navigate negativity online (34), less is known about how rural-living LGBTQ youth might leverage social media to have supportive experiences. The overarching goal of this study was to provide content ideas and guide development of an intervention to improve social media experience and reduce perceived isolation among rural-living LGBTQ youth. To this end, we examined their impressions regarding (1) supportive social media content and experiences, (2) personal strategies to improve their social media interactions and experiences, and (3) advantages and disadvantages of different delivery modalities for digital interventions seeking to improve social media experiences and reducing perceived isolation. We report the methods and results of this study according to the Consolidated Criteria for Reporting Qualitative Studies (COREQ) 32-item checklist (35), available in Appendix A (Supplementary Material).

## Methodology

### Sample Selection

From February through April 2020, we recruited LGBTQ adolescents living in rural areas of the United States using purposeful sampling via social media advertisements. This was appropriate given the purpose of our research (i.e., learning from the lived experience of LGBTQ youth living in rural areas who were using social media to combat isolation) and because purposeful sampling involves identifying and selecting participants that personally experience the phenomenon of interest (36). Given that LGBTQ youth are heavy social media users, (21) this medium allows for effective recruitment of participants from this diverse group, otherwise frequently underrepresented in research (37, 38). We placed ads on both Instagram and Facebook and utilized an ad creation feature to limit our ads to rural zip codes only according to the Federal Office of Rural Health Policy in the Health Resources Service Administration.

Interested youth completed an eligibility screener on Qualtrics (39) and those who were 14–19 years of age; screened positive for perceived social isolation; lived in a rural zip code; identified as gay, lesbian, bisexual, transgender, or queer; used at least one social media site; and spoke English were invited to an online interview. To screen for perceived social isolation, we used a 4-item measure developed by the Patient-Reported Outcomes Measurement Information System (PROMIS); (40) a score of 16 or more was considered positive (41). We obtained a waiver of parental consent to protect the privacy of participants under 18 years of age who might not have been out to their parents/guardians. Assent/consent forms included information about both the researcher and goal of the study. Participants downloaded a HIPAA compliant video conference application (42) to join the interview and received compensation for their time. All recruitment and data collection procedures were approved by the (University of Pittsburgh) Institutional Review Board.

### Data Collection and Interviews

Interviews were 60-min long and conducted by the first author, who has the background, research experience, and credentials for conducting individual interviews. We developed a structured interview guide (see Appendix B) based on both existing literature on the topic and our team's prior research related to social media, mental health, and LGBTQ people. Main topics included (1) supportive social media experiences; (2) personal strategies to improve social media experiences, and (3) pros and cons of different delivery modalities (e.g., mobile apps, chatbots, and website) for interventions focused on providing tips to rural-living LGBTQ youth for improving social media experience and reducing perceived isolation. Interviews were audio-recorded and transcribed verbatim. Transcriptions were then entered into NVivo 12 (43) for analysis.

### Analysis

We analyzed the data using a reflexive thematic analysis, in which we approached the data focused on semantic meaning, with an experiential and realist framework, as devised by Braun and Clarke (44–46). This was appropriate because it allowed us to identify and describe the themes driven by the data from LGBTQ youths' explicit meaning and perspectives about their lived experiences, (45, 46) and this matched the purposes of our study. The central unit of analysis was the participant. We used a hybrid deductive and inductive coding approach; this was appropriate because our coding framework was partly based on research on social media experiences among LGBTQ young adults, (47) but the lack of empirical evidence related to rural-living LGBTQ youth required us to provide meaning to concepts not clearly articulated in previous research (48). First, we randomly selected two transcriptions and created initial codes. Then, two co-authors used these codes as a template to double-code two additional transcripts. Code disagreements were resolved during team meetings. We used triangulation with the remaining co-authors (all of whom come from interdisciplinary fields of public health, medicine, and psychology and experience with qualitative research) to generate our final codebook which comprised 15 unique codes. After coding the remaining transcripts, we reviewed the codes searching for emerging themes, which we then redefined and named during team meetings.

## Results

Of 20 participants we interviewed, eight identified as cisgender gay/lesbian, three as cisgender bisexual, and nine as transgender or genderqueer. See Table 1 for demographic characteristics of participants. On the topic of supportive content and interactions, three themes emerged (1) positive representation of and connecting with LGBTQ groups on social media are important, (2) content from people with shared experiences feels supportive, and (3) lack of feedback to one's experiences reduces perception of support In relation with personal strategies to improve social media experiences, two themes emerged (1) selecting platforms to connect with different audiences helps make for a more enjoyable social media experience, and (2) several social media platform features can help make for a safer social media experience. Regarding perceived advantages and disadvantages of different delivery modalities for digital interventions, we present these results organized by the main delivery modality discussed by the participants. In all cases, we provide exemplar quotes from participants using pseudonyms to protect their privacy.

**Table 1:** Demographic characteristics of rural sexual and gender minority youth participants.

**Person^a^**	**Age**	**Birth**	**Gender**	**Sexual**	**Race/**	**State**
		**sex**	**identity**	**orientation**	**ethnicity**	
Hannah	18	Female	Cisgender	Bisexual	White	Missouri
Martin	16	Male	Cisgender	Gay	Black	Louisiana
Jackson	19	Female	Trans male	Bisexual	Pacific Islander	Nebraska
Paul	18	Male	Cisgender	Gay	White	Delaware
Grant	17	Male	Cisgender	Gay	White	West Virginia
Dustin	18	Male	Cisgender	Gay	White	Tennessee
Tony	17	Female	Trans male	Gay	White	Georgia
Cyrus	17	Male	Cisgender	Gay	White	Michigan
Adora	17	Female	Cisgender	Lesbian	White	California
Emily	19	Female	Cisgender	Bisexual	White	Michigan
Winter	14	Female	Genderqueer	Bisexual	White	New York
Payton	19	Female	Cisgender	Lesbian	White	Pennsylvania
Jason	15	Male	Genderqueer	Bisexual	White	Idaho
Alexi	18	Female	Cisgender	Lesbian	White	Maine
Skye	18	Female	Trans male	Bisexual	Hispanic	Georgia
Theo	17	Female	Trans male	Gay	Asian	Wisconsin
Elliot	19	Female	Trans male	Bisexual	White	North Carolina
Lily	19	Female	Genderqueer	Bisexual	White	Oklahoma
Adam	16	Female	Trans male	Bisexual	White	Washington
Allie	15	Female	Cisgender	Bisexual	White	Kansas

a *All names provided in the table are pseudonyms that were selected by the participants at the time the interviews were conducted*.

### Positive Representation of and Connecting With LGBTQ Groups on Social Media Are Important

Social media content and news that portray LGBTQ persons positively and in relatable ways, as well as readily available online resources, were considered key forms of support: “*When I was dealing with a lot of anxiety, I remember there were these ads on Facebook and Instagram that were like resources; I remember clicking on those and seeing a help line and useful information”* (Dustin, 18, gay, cis male). Youth said that even light content can be reassuring and supportive, if representation is positive: “*LGBTQ accounts with funny content make me smile, lift me up and I know that there are people out there rooting for me who wouldn*'*t want me to give up”* (Adora, 17, lesbian, cis female).

Participants thought LGBTQ groups that provide a sense of community were the most important support resource on social media: “*There*'*s a lot of good support groups on [Reddit], specific to certain people. There are many kids there… you can post, and people will give you advice, or support. I go to a couple sub-reddits that are for [bisexual] teens who are looking for support”* (Emily, 19, bisexual, cis female). Youth mentioned that connecting and building true friendships is an important function of these groups. One participant said that these groups allowed him to talk to people for years before meeting them in real life, which is valuable to him given the isolated area in which he lives. He added, “*Seeing the person physically is nice, but it doesn*'*t really matter [sic]. You can have a genuine connection and friendship with someone regardless of having physically met them or not.”* (Dustin, 18, gay, cis male). Another person said that having struggled with anxiety in the past, getting support and friends on social media groups was “*just easier”* for her “*because social media it*'*s non-confrontational, and usually people are supportive.”* (Hannah, 18, bisexual, cis female).

### Content From People With Shared Experience Feels Supportive

Posts from people with shared experience was mentioned as an important form of support on social media, and youth valued receiving support indirectly through hearing about the lived experiences of other LGBTQ people. Allie, a 15-year-old bisexual cis female, recounts her experience: “*When somebody writes something on social media that gets to me, I go to this [LGBTQ] group and just read about other people*'*s experiences and the feedback that other people had, and I can build a barrier against those mean comments I saw.”*

Youth mentioned that shared experience with other youth from rural areas was important: “*I relate more to suggestions from people that are in a rural community or have been in one. Whereas if they*'*re from a more accepting area, it*'*s harder to use their advice”* (Jackson, 19, bisexual, trans male). At the same time, they said anonymity allowed them to seek support through sharing meaningful details about themselves with others, while protecting their privacy: “*On Tumblr, I do not have a profile picture of me, but it has a lot more about me, my age, preferred name, pronouns, and my actual interests and beliefs”* (Lily, 19, bisexual, genderqueer).

### Lack of Feedback to One's Experiences Is Isolating

While social media was perceived as a positive tool for connecting with others and combatting isolation, LGBTQ youth identified specific forms of social media interactions that felt isolating. Participants mentioned seeing hurtful content, getting involved in online arguments, and being harassed on social media due to some of their profile content or posts. Most participants mentioned the inherent negativity of posting social media content that receives few or no feedback at all: “*It does sting when my posts are ignored by people and either not really looked at or just given a cursory reaction and then ignored”* (Lily, 19, bisexual, genderqueer). Some youth perceived that this lack of feedback assigned value to their attempts to reach out: “*I*'*ll take the time out to post a certain picture, like put some time into it, editing it and stuff. And then if it doesn*'*t receive as much reaction from people, that*'*s sometimes upsetting, not like a big deal, but it is annoying”* (Dustin, 18, gay, cis male). Lack of feedback to one's posts on social media also increased perceived isolation and disconnection: “*Probably the reason why I don*'*t like to post so much is because if I do and I don*'*t get any likes, I feel like there*'*s no one there”* (Cyrus, 17, gay, cis male). Youth described a learned process for coping with and improving these experiences, including the formation of a small network of close connections, and redefining the meaning of feedback on social media: “*Now I sort of have a core group of 5 or 6 people that kind of always see and like my posts and I realize not everybody that sees my things will like them. That does not define who sees me. It*'*s just who interacts with me”* (Emily, 19, bisexual, cis female).

### Selecting Platforms to Connect With Different Audiences Helps Make for a More Enjoyable Social Media Experience

Most youth mentioned they select specific platforms to connect with specific groups of people. For example, they would use different social media sites to connect with family members and classmates from school, with other LGBTQ persons, and to connect with online acquaintances or strangers. For some, this influenced whether to come out as LGBTQ on certain platforms: “*On Instagram I*'*m out because none of my family is on there. Facebook definitely not, because my family is there, and it is a small town and you can*'*t really avoid people on Facebook”* (Adam, 16, bisexual, trans male). This practice allows youth to go on social media and feel support from specific audiences: “*on Reddit, there*'*s people you can just talk to simply because they want to listen; on Instagram, I get support from close friends whom I know I can follow up later in real life”* (Jason, 15, bisexual, genderqueer).

### Several Social Media Platform Features Can Help Make for a Safer Social Media Experience

Participants described a vetting process to accept new friend/follower requests on social media. First, participants accept these requests only from people connected with persons they knew in real life, such as mutual real-life friends, family members, or well-known online friends. Next, “*scan their profile; take a look at them, see if they have what looks like a stock image photo and see what kind of details are available to me to see”* (Lily, 19, bisexual, genderqueer). Finally, checking biographic information and profile on other platforms, “*I might check those depending on what I find on the Instagram. But it*'*s, basically, do I see any content that makes me uncomfortable?”* (Tony, 17, gay, trans male).

With public accounts, participants managed their profiles using an alias or pictures not of themselves to filter unwanted requests. To prevent strangers from looking at their information, some preferred direct messaging with the person for a while first before accepting requests, and others chose to leave a new friend request or follower as pending. One said: “*If I know them, but I*'*m not really close, and I*'*m not open to sharing my things with them, I*'*d just either leave their request pending or else they*'*d know, and then they would talk to me in school about it”* (Winter, 14, bisexual, genderqueer).

For existing connections, adolescents used audience selection features to filter who would see their new posts: “*I blocked certain people from Instagram and Snapchat stories who just don*'*t see my posts about LGBTQ things”* (Alexi, 18, lesbian, cis female). If interactions with these connections were negative or unpleasant, they used the “block” option to reduce the likelihood of negativity, although they mentioned that sometimes, blocking also led them to a positive interaction later: “*It could be just a misunderstanding, or maybe they [people] just don*'*t know enough about LGBTQ topics and they just want to learn about something. I*'*ve had people just text me and say, ‘I know you posted about this. In the past, I didn't really agree with it, but I didn't know a lot about it. Could you just answer my questions about this?' I'm like, ‘Sure. Yeah”'* (Skye, 18, bisexual, trans male).

Youth used features such as voting up (e.g., giving a “thumbs up” to a post), hiding posts, or turning notifications off to determine the type of content they wanted to see on their feeds. Some curated content simply by choosing to connect with people interested in similar topics: “*On Instagram, it's either people that are similar to me or people who post art similar to my art,”* (Tony, 17, gay, trans male). To others, the best way to curate their feeds' content was to block, unfollow, or “take a break” from specific persons or groups: “*I curate my content based on who I want to talk to. When someone gives me bad vibes, I*'*ll just block them”* (Theo, 17, gay, trans male).

#### Perspectives on Different Technology-Based Intervention Delivery Modalities

Participants provided their perspectives on the potential advantages and drawbacks of different delivery modalities for digital mental health interventions for rural LGBTQ youth.

### Mobile App

An app to provide support resources and connect rural LGBTQ youth was a popular option. Participants noted a mobile app would be readily available on their phone and that would make them more inclined to use it. Martin (16, gay, cis male) mentioned the possibility that the app could detect when the user is sad and send uplifting content and notifications. Youth suggested the app could have a connection to a website or a social media functionality to connect with other LGBTQ youth and could allow tailoring of resources based on geography and identity. Another participant said an app “*has its own sense of anonymity as far as being monitored while using it”* (Paul, 18, gay, cis male). However, youth were concerned about the aesthetics and looks of the app; they said they would use the app only if it did not stand out too much on their phone as being LGBTQ-related, mainly out of fear of being outed to their parents/guardians. Some were skeptical about downloading yet another app with potentially few users (as opposed to an established social media platform), and said they tend to connect with people through their social media and did not think they would use another app for this.

### Social Media Pages/Groups

Youth mentioned closed groups and LGBTQ-specific pages on different social media platforms as a way of connecting with others. For many, closed groups were the only reason why they used some platforms, especially if family members were on these (i.e., Facebook). They suggested monitoring the interactions within these groups would be beneficial, and some suggested closed groups across several social media sites would be best to get younger LGBTQ people to join. Many mentioned their main concern is the number of negative interactions one can have. Hannah (18, bisexual, cis female) said, “*There are negative things I see in these groups about being bi, which are not true, but people post them loudly.”* To address this, youth suggested specific groups for youth of different gender or sexual identities. However, youth also said that either LGBTQ-specific pages or closed groups on social media would exclude LGBTQ youth who do not use that specific platform.

### Conversational Agent (I.e., Chatbot)

Youth were enthusiastic about using a chatbot to access well-curated, reliable LGBTQ-specific resources in their area, information about specific topics, and tailored to their identity; they stressed the importance of having these resources readily available for younger LGBTQ persons. They highlighted how a chatbot could help in easing the concerns of youth who feel they will receive judgement for asking questions about their identity, “*It would be better for my anxiety knowing that*'*s not a real person who*'*s going to judge me when they reply”* (Adora, 17, lesbian, cis female). Youth also suggested the chatbot could be deployed via an existing social media platform to both facilitate access to it and facilitate connections with others; this would address the inherent lack of human interaction. Nevertheless, several participants thought the chatbot was impersonal. Emily (19, bisexual, cis female) said, “*Sometimes you have to know what you*'*re feeling and how to say it in a way the bot will understand for it to give you the response you want”* and Jackson (19, bisexual, trans male) noted, “*Someone in a crisis could get disheartened to use something like that.”*

### Website

Some participants liked the option of a dedicated website providing support while offering easier ways of hiding one's online presence; they said this would be important in case parents/guardians are looking for apps or messages that could out the youth before they feel ready for it. Some mentioned a website would be distinct from a social media platform: “*It just feels different, whereas if you go on social media for it, you have the connotation of that platform, and it would also help giving you a sense of anonymity”* (Payton, 19, lesbian, cis female), and it would not require people to have a social media account. Adolescents mentioned the challenge of make for a website known to its potential audience; Tony (17, gay, trans male) suggested having a social media profile and linking it to the website, and Allie said online events, seminars, and providing LGBTQ-related news would help promote the site.

## Discussion

Social isolation in rural areas is prevalent and LGBTQ youth living in these areas are at compounded risk for perceived isolation and associated negative mental health outcomes, such as depression, anxiety, and suicidality (2, 7). Our findings provide insights on how rural-living LGBTQ youth who feel isolated manage their social media to gather supportive content and interactions, their strategies to do so, and their perspectives on potential delivery modalities for digital interventions seeking to enhance perceived social support among this understudied population.

In this sample of rural-living LGBTQ youth recruited from social media, most adolescents expressed they use it to connect with other LGBTQ youth and to seek both emotional and informational support. While emotional support has been strongly linked with mental health outcomes, (49) informational support may help increase self-efficacy for decision-making when facing big changes in one's life (50). Youth in our sample thought that social media spaces that provide a sense of community and content with a positive representation of LGBTQ people were important forms of support. This aligns with minority stress theory, which proposes that connecting with a like-minded community has a protective effect on mental health (51). These findings also support previous research on LGBTQ adolescents' development and the impact of positive representation on perceived social support (52).

Connecting with other LGBTQ people who share their often-intersectional identities and experiences (e.g., rurality, gender transitioning, exploring sexual identity, race, or ethnicity) seemed important for this sample of rural-living youth. These findings expand on previous research indicating that among developing LGBTQ youth, there is a strong preference towards LGBTQ-specific resources (11). On the one hand, this preference allowed them to establish connections and receive support through shared experiences with adolescents who were geographically distant. On the other hand, connecting with other LGBTQ people who lived elsewhere required means to remain anonymous to avoid potential perceived discrimination or rejection (e.g., maintaining different layers of anonymity, depending on the features available in each social media platform). These trade-offs rural LGBTQ youth felt the need to make in order to be able to connect with others like them on social media requires gaining awareness about potential negative online interactions and acquiring skills to block or evade these, and these are different from more traditional socializing processes such as connecting and developing friendships face-to-face (19). For example, while building meaningful connections on social media without in-person interactions is possible, users must adjust to a medium where the absence of non-verbal cues may force them to rely on limiting ways to express themselves and to interpret a given interaction, such as emojis or memes.

It is important to recognize the negative online interactions that youth in our sample identified, especially the lack of feedback to their social media posts and content as being particularly isolating. These findings complement and expand previous literature that found that sexual minority youth have smaller networks, respond less often when friends share good news, and join social media groups to make themselves feel less alone more often compared to their heterosexual peers (53). Insights from our study demonstrate that, among rural-living LGBTQ youth, attempts to cope with or to avoid expected rejection or discrimination might lead to creating very small networks and engaging with non-interactive browsing of social media, a form of passive usage, which has been associated with increased report of depressive symptoms among young adults (54). While this behavior may indeed reduce the opportunities for negative interactions, it may also lead to lost opportunities for establishing and growing new connections or nurturing offline friendships, potentially contributing to less perceived support and more isolation.

Our findings also identified strategies for optimizing their social media experiences that were important for rural-living LGBTQ youth. Most of these strategies focused on selecting specific platforms to connect with specific groups, using platform features for protecting their personal information, vetting profiles to identify potentially meaningful connections, and modifying their social media environment (e.g., home feeds) via content curation in ways that made them feel safer and more supported. These findings complement and expand previous research on strategies used by LGBTQ youth to cope with negativity online (34) by drawing from in-depth individual interviews to add the perspective of rural-living LGBTQ adolescents. On the one hand, our findings also suggest that to put these strategies into practice, youth also need reflective decision-making—for example, being comfortable with the decision to block a person with whom interactions are negative and unpleasant. Depending on personal characteristics and developmental stage, these processes might happen sooner for some youth than for others. On the other hand, it seemed almost contradictory that youth make decisions about who to engage with based on what they perceive to be real, but then they manage their own profile access by having pictures not of themselves and/or leaving information blank. While some of our participants mentioned that these strategies depend on the goals with which they use each specific social media site, this seems as if it would be problematic, as they would not interact as themselves based on their profiles. Research is needed to explore both this seeming discordance and the feasibility of leveraging these strategies and skills into potential support interventions to assist LGBTQ adolescents living in rural areas to optimize their social media experience.

Our findings also shed a light regarding the potential use of several delivery modalities for providing support and reducing perceived isolation among rural-living LGBTQ youth, including mobile apps, stand-alone websites, social media support groups, and conversational agents. While youth had positive attitudes toward the usage of different technology-based delivery modalities, combining these modalities (as opposed to using them separately) were favored by youth in our sample recruited from social media. Importantly, combining delivery modalities for behavioral interventions might provide a way to increase intervention uptake and engagement. This finding aligns with previous research indicating that digital intervention users prefer information and other content delivered in more than one way of interactivity (i.e., user interface) (33).

Youth identified advantages for every proposed delivery modality. These included the ready availability of a mobile app; the potential of using closed groups on social media to get support from people with shared experience; the potential utility of a chatbot for providing tailored, LGBTQ-specific support and resources; and the opportunity of using a website to serve youth who are not on social media or who want more privacy. These suggested uses underscore the importance of selecting a delivery modality that allows tailoring and fitting intervention content, activities, and goals with those of the potential end user, which is one of the key factors to increase engagement with digital interventions (33). However, youth in our sample also expressed concerns with each of the delivery modalities, including the visual interface of a mobile app, a potentially low number of users on a mobile app, the potentially high number of negative interactions on social media, likelihood of impersonal interactions with a chatbot, and challenge of promoting a website to its potential audience. While some of these concerns also speak to the importance of tailoring interventions to the specific user needs and goals, these concerns also highlight that delivery modalities for digital interventions might be more engaging when they incorporate human support in some way (33). This human support could include actual opportunities for connecting with other users, peer support, and learning through vicarious experience.

Finally, our findings echo and expand previous research about how to promote engagement with digital interventions adding specific input from rural-living LGBTQ youth. Digital technology holds potential for increasing the reach and immediacy of educational interventions, self-management of well-being, and delivery of mental health services, as well as promoting community support and resilience among LGBTQ people in rural areas. While access to both broad band and smartphones are still lower among rural-dwelling people than those living in urban or suburban areas, smartphone ownership among rural residents has increased dramatically and it is closing the gap with suburban dwellers (55). Given that engagement with the intervention is a pre-requisite for effectiveness (25, 60), it is crucial that development, adaptation, optimization and scaling of digital interventions for LGBTQ adolescents living in rural areas are conducted with the input, feedback, and active participation of youth with lived experience in order to increase the likelihood of users engaging with and using the interventions.

## Limitations

Our study is not without limitations to consider. First, while we recruited a diverse sample in terms of geographic regions, gender identity, and sexual identity, participants were predominantly White. We tailored our social media ads to specific racial and ethnic identities by including recruitment images featuring people of color in hopes of making the ads more appealing. Despite these efforts, we were unable to increase our recruitment of these youth. Therefore, we had limited ability to capture the impressions at the intersection of LGBTQ, racial and ethnic identities. However, ours is one of few qualitative studies examining the perceptions of rural-living LGBTQ adolescent users of social media about their experiences with this medium, as well as behaviors and strategies they implemented to foster positive experiences, and delivery modalities that would be useful for a technology-based intervention focused on increasing support to reduce perceived isolation. Second, because we recruited using social media, we did not capture the experience with social isolation of LGBTQ youth that did not use social media. While social media use among youth is fairly ubiquitous, (19) access to broadband and smartphones in rural America is still lower (roughly 72 and 80%, respectively) than urban (77 and 89%, respectively), and suburban areas (79 and 84%, respectively) (55). Nevertheless, previous research indicated that when LGBTQ youth go online to combat isolation and to find the support their offline environment frequently lacks, they also encounter negative interactions that might increase feelings of isolation and loneliness. Our recruitment of LGBTQ youth users of social media who felt isolated helped us to learn from the experiences of this group to inform potential interventions and delivery modalities best suited for this subgroup of youth.

## Conclusions

Rural-living LGBTQ youth who feel socially isolated and use social media turn to several of these platforms to seek support and to connect with others like them in meaningful ways. The results of our study inform potential intervention targets and delivery modalities for intervention development focused on reducing perceived isolation among this group of youth. Viewing positive representation of and connecting with LGBTQ groups, content from people with shared experiences, and utilizing a wide array of platform features to increase the likelihood of positive connections are key to a positive social media experience among this group. Combining delivery modalities is key to engaging rural-living LGBTQ youth in digitally delivered support interventions to reduce perceived isolation. While these findings may inform future intervention research, the results of this study might be useful for clinicians serving in rural areas who seek to initiate a conversation with their LGBTQ patients about the influence of their social media experience on mental health as part of their assessment of social determinants of health.

## Data Availability Statement

The raw data supporting the conclusions of this article will be made available by the authors, without undue reservation.

## Ethics Statement

The studies involving human participants were reviewed and approved by Human Research Protection Office (HRPO) at the University of Pittsburgh.

## Author Contributions

CE-V conceptualized, designed the study, conducted the qualitative interviews, developed the initial codebook and themes, and drafted the initial manuscript. SC-B and JS contributed to developing the codebook, themes, and provided edits and feedback to the manuscript. AM and SR coded the data, contributed to developing the themes, and provided edits and feedback to the manuscript. BR contributed to conceptualizing the study and developing the themes, and provided edits and feedback to the manuscript. All authors contributed to the article and approved the submitted version.

## Funding

We gratefully acknowledge funding from NIMHD (Grant Number R00—MD012813) and NIMH (Grant Number P50—MH115838) for the completion of this research. Contribution from AM and SR was supported with funding from NSF (Grant Number 1940700). Additionally, this work was supported by the Clinical and Translational Science Institute (CTSI) at the University of Pittsburgh. The CTSI at the University of Pittsburgh is supported by the National Institutes of Health (NIH) Clinical and Translational Science Award (CTSA) program, grant UL1 TR001857. Funding sources played no role in the study design, collection, analysis, interpretation of data, writing of the report, or decision to submit the article for publication.

## Conflict of Interest

The authors declare that the research was conducted in the absence of any commercial or financial relationships that could be construed as a potential conflict of interest.

## Publisher's Note

All claims expressed in this article are solely those of the authors and do not necessarily represent those of their affiliated organizations, or those of the publisher, the editors and the reviewers. Any product that may be evaluated in this article, or claim that may be made by its manufacturer, is not guaranteed or endorsed by the publisher.
