# Supplementary material for: Examining Social Media Experiences and Attitudes Toward Technology-Based Interventions for Reducing Social Isolation Among LGBTQ Youth Living in Rural United States: An Online Qualitative Study
Source: Front Digit Health. 2022 Jun 27;4:900695. doi: 10.3389/fdgth.2022.900695 (PMC9271672; doi:10.3389/fdgth.2022.900695)
Supplement: Supplementary file 1 [file Table_1.DOCX]

| Appendix A. Consolidated criteria for reporting qualitative research (COREQ) Table |  |
| --- | --- |
| COREQ Reporting Criteria | Manuscript page |
| **Domain 1: Research team and Reflexivity** |  |
| Study clearly states which authors conducted the interview/focus group | 6 |
| Credentials of the researchers are evident | 6 |
| Researcher occupation at the time of the study is reported | 6 |
| The gender of the researcher(s) is reported | 6 |
| The experience and training of the researcher is reported | 6 |
| Study reports if a relationship with participants was established before study commencement | 5 |
| Study reports what, if anything, participants knew about the researcher before the study commencement | 5 |
| Study reports any interviewer characteristics such as bias or assumptions | 6 |
| **Domain 2: Study Design** |  |
| Study states the methodological orientation and theory underpinning the study | 4 |
| Study explains the methods of participant selection | 5 |
| Study explains the method of participant approach | 5 |
| Study reports and explains the sample size included | 5 |
| Study explains reasons for participation refusal and attrition | 5 |
| Study explains setting of data collection | 5 |
| Study reports any non-participants present during data collection | 5 |
| Study reports demographic information about participants | Table 1 |
| If a guide was used, the researcher explains guide development and pilot testing | 6 |
| If repeat interviews were given, the study reports the number and reason | Not applicable |
| Study reports any audio/visual recording methods of data collection | Not applicable |
| Study reports if field notes were made during or after the focus group(s) | Not applicable |
| Study reports the duration of focus group(s) and interview(s) | 6 |
| Study reports if data saturation was reached | 8 |
| Study reports if transcripts were reviewed by participants for comment or correction |  |
| **Domain 3: Analysis and Findings** |  |
| Study reports the number of data coders | 7-8 |
| Researchers provided a description of the coding tree | 7-8 |
| Researchers explained if themes were predetermined or derived from data | 7-8 |
| Researchers reported what software, if any, was used in data analysis | 7 |
| Researchers reported on the use of member checking | 7-8 |
| Participant quotations, attributed to specific participants, were included to demonstrate themes | 9-16 |
| Study reports findings consistent with the data | 17-21 |
| Study presents clear themes | 9-16 |
| Study presents the diversity of cases and any minor themes determined from data | 9-16 |

Note: COREQ criteria extrapolated from Tong A, Sainsbury P, Craig J. Consolidated criteria for reporting qualitative research (COREQ): a 32- item checklist

for interviews and focus group*. Int J Qual Heal Care*. 2007;19(6):349-357. doi:10.1093/intqhc/mzm042.
