# Supplementary material for: Examining Social Media Experiences and Attitudes Toward Technology-Based Interventions for Reducing Social Isolation Among LGBTQ Youth Living in Rural United States: An Online Qualitative Study
Source: Front Digit Health. 2022 Jun 27;4:900695. doi: 10.3389/fdgth.2022.900695 (PMC9271672; doi:10.3389/fdgth.2022.900695)
Supplement: Supplementary file 2 [file Table_2.DOCX]

INDIVIDUAL INTERVIEW GUIDE

*Consistent with qualitative methodologies, wording and order of questions may be modified based on participant lead and ongoing analyses.*

Hi, my name is [redacted for review] and I will conduct our interview today. We are doing this research to learn how we might use social media to help lesbian, gay, bisexual, transgender, non-binary, and other queer kids combating social isolation. During the interview, you can use a made-up name, or you can use your preferred name, whichever you prefer. How do you want me to call you during our conversation?

Some of the questions touch may touch on sensitive topics, and might make you feel a bit uncomfortable. If you feel that the interview is becoming too stressful for you, all you have to do is to tell me you want to end the interview. This is OK and you still will be compensated for your participation. Do you have any questions for me before we start?

I will start recording the interview now. Let’s get started!

Engagement Questions

- Tell me about how you socialize and make friends these days.
  - How about socialize and make friends with other LGBTQ kids?
- Describe to me what comes to mind when you hear the term social media.
  - What is your general opinion about social media?
- Tell me about your reasons for using social media.

Exploration Questions

- In our survey, you mentioned your 3 favorite social media apps are ­­­­­­­­­­_______, _______, and _______. For the next questions, I want you to answer them specifically thinking about these apps. Is that OK?
- I would like to know more about the way you manage your profile on (mention 3 sites)
- Now, I want to ask you about the way you handle the people you connect with on (mention 3 sites)
  - **Probe** how they choose to accept a friend request
  - Do these choices differ across (mention 3 sites)? IF YES, in which way? IF NO, move on
- People have different experiences when they go on social media. For example, some people join groups that make them feel like they belong, but some might feel ignored when their posts do not receive enough reactions/likes. Tell me about the experiences you had on social media.
- Some people go to social media to seek support when they are going through a hard time (e.g., feeling isolated or down). Tell me about your experience seeking support from social media.
  - **Probe** favorite app to seek/receive support, how is support provided in this app
- We have some ideas about how to provide support to other LGBTQ kids living in rural areas. I want to hear your feedback on these ideas, and more importantly, hear your own ideas about these.
  - Instagram page
  - Facebook closed group
  - Dedicated website
  - Chatbot
  - Mobile app

Exit Question

- Is there anything else you would like to say about how social media could be used to help LGBTQ kids combating social isolation?
